# Molecular characterization of chicken astrovirus and pathogenicity of a novel isolate in China

**DOI:** 10.3389/fmicb.2023.1280313

**Published:** 2023-12-11

**Authors:** Xiaofeng Han, Lijuan Yin, Xiaoying Liang, Huazhen Liang

**Affiliations:** Wen’s Foodstuffs Group Co., Ltd., Yunfu, Guangdong, China

**Keywords:** chicken astrovirus, molecular characterization, isolation, phylogenetic analysis, pathogenicity

## Abstract

As an enteric virus, chicken astrovirus has been related to various kinds of diseases in chickens, including white chick syndrome, runting-stunting syndrome, severe kidney disease, urate deposits and visceral gout, generating economic losses in the poultry industry globally. The complete ORF2 gene of 31 CAstV isolates in six provinces of China during 2020–2022 was characterized and analyzed with the purpose of better understanding the molecular epidemiology and genetic diversity of CAstV field isolates. Phylogenetic analysis which was based on the complete ORF2 (capsid) amino acid sequence of 31 CAstV isolates and 57 reference strains indicated that 2 isolates belonged to subgroup Ai, 10 isolates belonged to subgroup Bi, 3 isolates belonged to subgroup Bii, 5 isolates belonged to subgroup Biii, 7 isolates belonged to subgroup Biv, 3 isolates belonged to subgroup Bv, and one isolate (JS202103) belonged to a new B subgroup. In addition, the novel CAstV strain JS202103 was successfully isolated *in vitro*, and its whole genome shared 76.9–94.3% identity with the 29 CAstV reference strains. JS202103 caused hatchability reduction, dead embryos, kidney disease and visceral gout in chicken embryos. Moreover, this is the also the initial study focusing on diverse CAstV strains including subgroups Biii, Biv, and Bv circulate in China. The current work contributes to improving our understanding of CAstV isolates in China, and it will also provide references for developing efficient measures to control this virus.

## Introduction

Chicken astrovirus (CAstV), belonging to genus Avastrovirus in Astroviridae family, acts as an etiologic agent of enteric disease in chickens, and makes a primarily effect on the poultry industry, and generates economic losses worldwide (Raji and Omar, 2022a,b). CAstV is a star-shaped morphology (28–30 nm in diameter), non-enveloped, positive-sense, and single-stranded RNA virus (Smyth, 2017; Raji and Omar, 2022a,b). The around 7.5 kb genome includes one 5′-untranslated region (5’-UTR), one 3′-untranslated region (3’-UTR), and three open reading frames (ORFs), which encode two non-structural proteins designated as trypsin-like serine protease within ORF1a, the RNA-dependent RNA polymerase within ORF1b, and a structural capsid protein within ORF2 (Kang et al., 2012; Cortez et al., 2017). The chicken astrovirus genome is arranged in the 5’UTR-ORF1a-ORF1b-ORF2-3’UTR order (Kang et al., 2012). As the most divergent region of the whole genome, the ORF2 gene encodes one surface protein of viral particles and is accountable for the virus antigenicity, cell tropism, pathogenicity, and immune protection (Smyth et al., 2012; Smyth, 2017). According to the capsid protein, CAstV strains can be categorized into two distinct genogroups: Group A (three subgroups: Ai, Aii, and Aiii) and Group B (six subgroups: Bi, Bii, Biii, Biv, Bv, and Bvi) (Smyth, 2017; Raji et al., 2022; Raji and Omar, 2022a,b).

CAstV was initially identified from broiler flocks with uneven growth and runting-stunting syndrome in the Netherlands in 2004 (Baxendale and Mebatsion, 2004), and then emerged in many countries globally (Pantin-Jackwood et al., 2011; Smyth, 2017; Xue et al., 2017, 2020; Raji and Omar, 2022a,b). CAstV is transmitted both vertically and horizontally and can often infect chickens at an early age (Koci and Schultz-Cherry, 2002; Smyth, 2017). CAstV infection has been correlated with numerous diseases, such as white chick syndrome (WCS) or white chick hatchery disease, runting-stunting syndrome (RRS) or uneven growth, locomotion and enteric diseases, and kidney disease with visceral gout, posing great threats to the poultry industry (McIlwaine et al., 2021; Li et al., 2022). White chick syndrome (WCS), featured by reduced egg yield and hatchability or a mid-to-late death-in-shell embryo in susceptible breeders, as well as small-size, weakness, pale plumage, and distended abdomen in young hatched broilers, has been reported in a lot of countries, such as the United Kingdom, United States, Canada, Brazil, Poland, Norway and Finland (Smyth et al., 2013; Sajewicz-Krukowska et al., 2016; Long et al., 2017; McIlwaine et al., 2021). Runting-stunting syndrome (RSS) has been reported to cause maldigestion, malabsorption and enteritis, stunting and ruffled feathers, retardation, and uneven flock performance in very young broilers, resulting in a high culling rate and decrease in turnover (Baxendale and Mebatsion, 2004; Kang et al., 2012). In addition, CAstV infection has also been correlated with severe kidney disease, urate deposits and visceral gout, circulating in India, Malaysia, and the Middle East (Bulbule et al., 2013; Smyth, 2017; Raji et al., 2022).

Since 2016, CAstV infections have been detected circulating in China (Xue et al., 2017; Zhao et al., 2020; Yin et al., 2021; Luan et al., 2022). For example, Xue et al. did first serological investigation of CAstV infection and showed that CAstV infection is very common in China (Xue et al., 2017). Zhao et al., first isolated a CAstV strain (CHN/2017/NJ1701, subgroup Bi) causing stunted embryo and significant decrease in hatching rate to SPF chicken embryos (Zhao et al., 2020). Yin et al., isolated a novel and recombinant CAstV strain (GD202013, subgroup Bii) leading to enteritis, swollen kidney, enlarged liver with numerous hemorrhages, and abscission in small intestines in SPF chicken embryos (Yin et al., 2021). Luan et al., isolated three types of genotypic CAstV (subgroup Ai, Bi and Bii), resulting in high mortality in SPF chicken embryos and leading to WCS and visceral gout in SPF chickens (Luan et al., 2022).

We molecular characterized the 31 CAstV isolates from six provinces of China in 2020–2022. A novel CAstV strain JS202103 was successfully isolated and serially cultured in LMH cell culture to investigate its pathogenicity to specific pathogen-free chicken embryos. In addition, for the purpose of comparing the novel CAstV strain (JS202103) with other reference strains, we adopted sequence alignment, phylogenetic as well as recombination analyses. Moreover, the present study can also provide the basis better understanding the genetic evolution and molecular pathogenesis of chicken astrovirus in China.

## Materials and methods

### Sample collection and virus detection

During the period of 2020–2022, several 1 day-old chicken flocks with poor viability of hatched chicks, diarrhea and growth problem were suspected of being infected with CAstV. A total of 69 samples, including kidneys, livers, and intestines, were collected from the suspected chicken flocks from six provinces of China, including Guangdong, Fujian, Jiangxi, Jiangsu, Anhui, and Shandong. Tissue samples were prepared as 10% weight/volume homogenates in phosphate-buffered saline (PBS) with 1% antibiotic-antimycotic (Gibco, United States). Following the instructions of the manufacturer, the nucleic acid was extracted based on the MiniBEST Viral RNA/DNA Extraction Kit (Takara, Dalian, China). In line with the previous description (Smyth et al., 2010), a CAstV ORF1b gene-based real-time polymerase chain reaction (PCR) was conducted to show whether CAstV exists in samples. The existences of other pathogens, which included fowl adenovirus (FAdV), Newcastle disease virus (NDV), avian nephritis virus (ANV), avian reovirus, infectious bronchitis virus (IBV), chicken parvovirus (ChPV), and avian rotavirus, were evaluated by real-time PCR with primers (Supplementary Table S1) and methods as previously described (Wise et al., 2004; Callison et al., 2006; Smyth et al., 2010; De la Torre et al., 2018; Nunez et al., 2018; Li et al., 2020).

### RT-PCR, cloning, and nucleotide sequencing

In line with the guidance of the manufacturer, the viral RNA of CAstV isolates was isolated with the MiniBEST Viral RNA/DNA Extraction Kit (Takara, Dalian, China). Using PrimeSTAR Max Premix (Takara, Dalian, China) as previously described (Zhao et al., 2020), the whole ORF2 gene of the 31 CAstV isolates was amplified. Additionally, as previously described (Smyth et al., 2009; Zhao et al., 2020), the whole genomic sequence of the CAstV isolate JS202103 was amplified by 7 pairs of primer synthesized with conserved regions of CAstV sequences. All gel-purified PCR products were inserted in a pMD18-T vector (TaKaRa, Dalian, China). Three or more positive clones for every PCR fragment were selected for DNA sequencing in Sangon Biotech Company (Guangzhou, China). All nucleotide sequences generated in the present study were submitted to GenBank.

### CAstV isolation, plaque purification, and propagation in LMH cells

Only CAstV-positive tissue samples were used to isolate CAstV. Virus isolation, propagation, and purification of CAstV were attempted on leghorn male hepatoma (LMH) cells (ATCC #CRL-2117) according to previous description (Baxendale and Mebatsion, 2004; Zhao et al., 2020). In brief, LMH cell monolayers were seeded within the 12-well plates, kept with Dulbecco’s Modified Eagle’s Medium (DMEM) medium (Hyclone, Logan, Utah, United States) that contained 10% fetal bovine serum (FBS) (BOVOGEN, Australia) as well as 1% antibiotic-antimycotic (Gibco, Grand Island, NY, United States), and subjected to incubation at 37°C with 5% CO_2_. The CAstV-positive suspension was filtered via the 0.22 μm-pore-size syringe filter and inoculated into monolayers of LMH cells. Until the obvious cytopathic effects (CPEs) were found, both cells and supernatant were collected through three freeze-thaw cycles for the subsequent virus propagation, plaque purification, as well as real-time PCR analysis (Smyth et al., 2010). In terms of plaque purification of the CAstV strain, LMH cells were inoculated into the 6-well plates at 80% confluence. After being subject to incubation for 18 h, the medium was removed and infected with 500 μL of a 10-fold dilution series of virus suspension. Following incubation of 1 h at 37°C with 5% CO_2_, the cells were replaced with 2 mL DMEM medium including 1% antibiotic-antimycotic and 1% Agarose LM GQT (TaKaRa, Dalian). To solidify the overlaid medium, the 6-well plates were subject to incubation at 4°C for 30 min. Subsequently, the cells were grown at 37°C with 5% CO_2_ to form plaque. Plaques were stained with neutral red solution (Sigma, United States) at 3–5 days post-inoculation, followed by picking and propagation within 12-well plates till CPE became obvious. Viral propagation was performed using the selected plaques. A procedure described previously was used to carry out the 50% tissue culture infective doses (TCID_50_) assay (Kang et al., 2018).

### Immunofluorescence assay

The indirect immunofluorescence assay (IFA) was made in line with previous description with slight modifications (Kang et al., 2018; Zhao et al., 2020). Briefly, CAstV (multiplicity of infection (MOI), 0.1) was added to incubate LMH monolayers within 6-well plates for a 2 days period. After fixation within 4% paraformaldehyde supplemented in PBS, cells were subjected to permeabilization using 0.5% Triton X-100 prior to blocking by 2% bovine serum albumin (BSA). In this study, CAstV specific mouse antisera (Wen’s Foodstuffs Group Co., Ltd., China) (dilution, 1:200) was applied as primary antibody. Thereafter, Cy3-conjugated goat anti-mouse IgG secondary antibody (Beyotime, Shanghai, China) (dilution, 1:500) was adopted to incubate cells. Later, 4′-6′-diamidino-2-phenylindole (DAPI, 10 μg/mL) was introduced in nuclear staining. In addition, a fluorescence microscope (IX73, Olympus, Japan) was used for visualizing the cells.

### Sequence comparison, phylogenetic analysis, and recombination analysis

Seqman program of DNAstar Lasergene 7.1 software (DNAStar, Madison, WI, United States) was used to assemble the seven overlapping sequences of the CAstV strain in the complete genome sequence. In addition, Clustal W method in the Megalin program of DNAstar Lasergene 7.1 software (DNAStar, Madison, WI, United States) was used to perform the sequence alignments. The phylogenetic trees on the basis of whole genome, ORF1a, ORF1b, and ORF2 amino acid sequence were constructed by adopting neighbour-joining method of MEGA 7.0 software with 1,000 bootstrap replicates (Kumar et al., 2016). In addition, to determine those potential recombination events of CAstV strain JS202103, we explored aligned sequences with recombination detection program (RDP) software version 5.5 (Martin et al., 2015), and Simplot software version 3.5.1 (Lole et al., 1999).

### Pathogenicity test of CAstV JS202103 strain in SPF chicken embryos

According to the previous description with minor modifications (Zhao et al., 2020; Yin et al., 2021), the pathogenicity of cell-cultured CAstV strain JS202103 in passage four was revealed in chicken embryos. Specific pathogen-free (SPF) eggs applied in the present study were obtained from Guangdong Wens Dahuanong Biotechnology Co., Ltd., China. A total of sixty 9 days-old SPF embryonated chicken eggs were randomly and equally classified into two groups (*n* = 30 eggs/group). All chicken embryos were inoculated with 0.2 mL of DMEM including 5.13 × 10^4^ TCID_50_/mL CAstV JS202103 strain through the yolk-sac route, as shown in the experimental group. DMEM (0.2 mL/egg) was introduced for inoculation in control group by the same route. Afterwards, all chicken embryos were returned into an egg incubator at 37°C with 55% relative humidity, rotated till day 21 during embryonic development, observed for the viability of the embryos every 24 h after inoculation, and the dead embryos suspected of bacterial contamination in the first 24 h were discarded. Mortality rate was recorded and dead embryos were necropsied. After the necropsy, the fresh samples (allantoic fluid, heart, liver, kidney and intestine) were gathered. Using real-time PCR, one portion of sample was used to analyze virus distribution, while the other portion was fixed with 4% formaldehyde, prepared in the 4 μm sections, and stained using hematoxylin and eosin (HE). Then, the observation was made under light microscopy for histopathology analysis.

### Quantitative real-time RT-PCR analysis

In accordance with the previous description (Smyth et al., 2010), allantoic fluid and various tissues were examined through a CAstV ORF1b gene-based real-time PCR which included viral standards with known plasmid content for quantification. In brief, specific primer sets and probes for conserved regions in ORF1b of CastV genome were synthesized by Sangon Biotech (Guangzhou, China). For the purpose of performing the real-time PCR assay, we adopted Applied Biosystem 7,500 Fast Real-Time PCR System (Thermo Fisher, United States). Based on the instructions of the manufacturer, Premix Ex Taq^™^ (Probe qPCR) (Takara, Dalian, China) was used to perform the real-time PCR. The conditions of amplification were presented: 95°C for 20 s, 40 cycles of 95°C for 3 s, and 60°C for 30 s.

### Statistical analyses

Every experiment was carried out thrice in an independent way, which yielded similar results. In order to analyze whether inter-trial variability was significant, we used GraphPad Prism software version 6 in this study. Data were indicated to be mean ± standard deviations (SD). For the purpose of performing all the statistical analyses, we adopted student’s *t* test. All the existing differences between groups were thought to be of statistical significance at *p* < 0.05.

## Results

### Detection of CAstV from clinical samples

**Figure 1:**
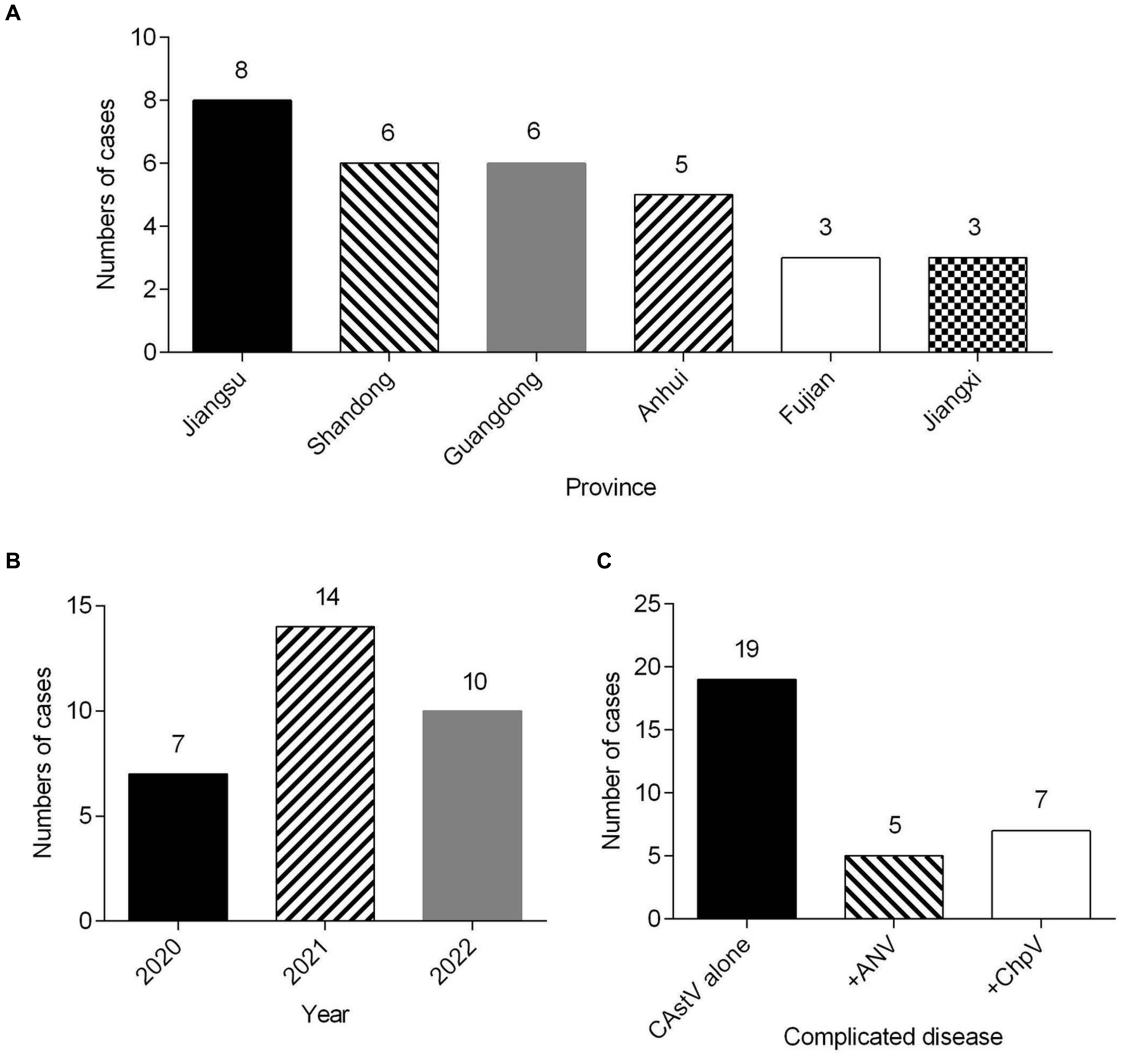
Clinical cases (*n* = 31) of CAstV infections in 1 day-old chicken flocks between 2020 and 2022 in China. **(A)** Geographical distribution of clinical cases of CAstV. **(B)** Temporal distribution of clinical cases of CAstV. **(C)** The presence of other viral pathogens.

From 2020 to 2022, totally 69 clinical samples from 1 day-old chicken flocks with poor viability of hatched chicks, diarrhea and growth problem were detected for CAstV by real-time PCR. Among them, 31 (44.9%) clinical samples were positive for CAstV, and epidemiological data of 31 CAstV isolates were depicted in Table 1. Spatial analysis demonstrated that CAstV isolates appeared in six provinces, and 8 CAstV isolates were identified in Jiangsu province (Figure 1A). Temporal analysis indicated that the number of CAstV isolates gathered from 2020 to 2022 was 7, 14, and 10, respectively (Figure 1B). Additionally, this study selected co-infections with additional viral pathogens, including avian reovirus, avian rotavirus, ChPV, ANV, NDV, IBV, and FAdV. For these pathogens, 19 cases were only positive for CAstV, five cases were positive for CAstV and ANV, seven cases were positive for CAstV and ChPV, and none was positive for avian reovirus, avian rotavirus, NDV, IBV and FAdV (Figure 1C).

**Table 1:** Epidemiological findings of chicken flocks positive for CAstV in China.

No.	Accession No.	Isolate	Province	Year	Length	Subgroup	Pathogens
1	OR359230	AH202057	Anhui	2020	2,163 bp	Ai	CAstV, ChPV
2	OR351272	AH202016	Anhui	2020	2,232 bp	Bii	CAstV
3	OR359211	JS202034	Jiangsu	2020	2,232 bp	Bii	CAstV, ANV
4	OR359212	JS202015	Jiangsu	2020	2,232 bp	Bii	CAstV
5	OR359229	SD202005	Shandong	2020	2,157 bp	Ai	CAstV
6	OR359202	SD202003	Shandong	2020	2,217 bp	Biv	CAstV, ChPV
7	OR359228	SD202050	Shandong	2020	2,217 bp	Bv	CAstV
8	OR359224	AH202101	Anhui	2021	2,217 bp	Biii	CAstV, ChPV
9	OR359223	AH202107	Anhui	2021	2,217 bp	Biii	CAstV
10	OR359222	AH202155	Anhui	2021	2,217 bp	Bv	CAstV
11	OR359220	FJ202132	Fujian	2021	2,214 bp	Bi	CAstV, ChPV
12	OR359221	FJ202110	Fujian	2021	2,214 bp	Bi	CAstV
13	OR359208	JS202109	Jiangsu	2021	2,217 bp	Biii	CAstV, ANV
14	OR359210	JS202104	Jiangsu	2021	2,217 bp	Biv	CAstV
15	OR359209	JS202106	Jiangsu	2021	2,217 bp	Biv	CAstV, ChPV
16	OR351273	JS202103	Jiangsu	2021	2,217 bp	unknown	CAstV
17	OR359204	JX202140	Jiangxi	2021	2,214 bp	Bi	CAstV
18	OR359205	JX202104	Jiangxi	2021	2,214 bp	Bi	CAstV
19	OR359226	SD202103	Shandong	2021	2,217 bp	Biii	CAstV
20	OR359225	SD202106	Shandong	2021	2,217 bp	Biv	CAstV, ANV
21	OR359227	SD202101	Shandong	2021	2,217 bp	Bv	CAstV
22	OR359219	FJ202270	Fujian	2022	2,214 bp	Bi	CAstV, ChPV
23	OR359215	GD202268	Guangdong	2022	2,217 bp	Bi	CAstV
24	OR359217	GD202232	Guangdong	2022	2,217 bp	Bi	CAstV
25	OR359213	GD202282	Guangdong	2022	2,217 bp	Biii	CAstV
26	OR359216	GD202234	Guangdong	2022	2,217 bp	Biv	CAstV, ANV
27	OR359218	GD202224	Guangdong	2022	2,217 bp	Biv	CAstV
28	OR359214	GD202277	Guangdong	2022	2,217 bp	Biv	CAstV, ChPV
29	OR359203	JX202227	Jiangxi	2022	2,214 bp	Bi	CAstV
30	OR359206	JS202254	Jiangsu	2022	2,214 bp	Bi	CAstV, ANV
31	OR359207	JS202231	Jiangsu	2022	2,214 bp	Bi	CAstV

### Viral isolation and identification

**Figure 2:**
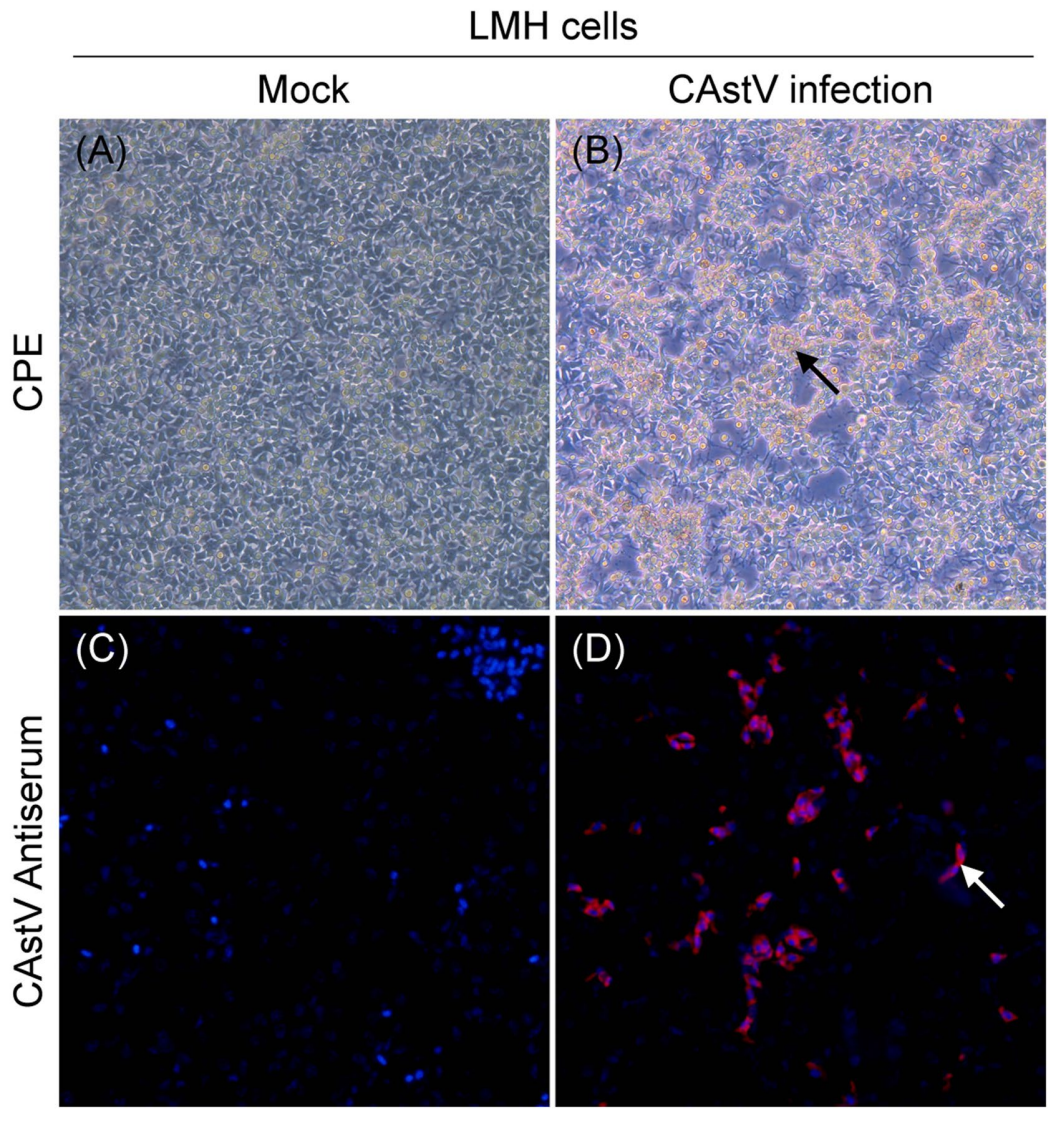
Virus isolation and identification of the CAstV strain JS202103 in LMH cells. **(A)** LMH cells control. **(B)** CPE formation in LMH cells infected with the JS202103 showing as detached small and round at 48 h post-infection (black arrow). **(C,D)** LMH cells were fixed at 48 h post-infection, and incubated with CAstV-specific mouse antisera followed by incubation with Cy3-conjugated goat anti-mouse secondary antibody (red fluorescence). Then, the cells were nuclear stained with DAPI (blue fluorescence).

We attempted to isolate chicken astrovirus from the 19 only CAstV-positive clinical samples using LMH cells. One CAstV isolate, named JS202103, was isolated from one kidney tissue sample gathered from a chicken flock with growth problem and poor viability of hatched chicks in Jiangsu province. A clear CPE was featured by the detached small and round cells at the beginning of passage three (Figure 2B) in relative to the control LMH cells (Figure 2A), like CPE of CAstV-infected cells (Kang et al., 2018; Zhao et al., 2020). It was further confirmed by CAstV-specific real-time PCR targeting as the ORF1b gene. The JS202103 strain was only positive for CAstV whereas negative for additional frequently seen enteric viruses such as avian reovirus, ANV, IBV, FAdV, avian rotavirus, NDV, and ChPV by real-time PCR. After plaque purification and four passages, viral titer was 5.13 × 10^6^ TCID_50_/mL, suggesting that CAstV JS202103 strain showed high replication within LMH cells. IFA results showed that red fluorescence was just found in CAstV-infected LMH cells (Figure 2D), compared with control LMH cells (Figure 2C).

### Sequence comparison and phylogenetic analysis of ORF2 genes of CAstV

The 31 ORF2 gene sequences of CAstV obtained in the present study have been deposited in GenBank (Table 1). The 18 ORF2 genes identified were 2,217 bp in length, the 8 ORF2 genes identified were 2,214 bp in length, the 3 ORF2 genes identified were 2,232 bp in length, the ORF2 gene of CAstV AH202057 was 2,163 bp in length, and the ORF2 gene of CAstV SD202005 was 2,163 bp in length. Nucleotide identity of 48.8–99.9% and amino acid (aa) identity of 38.8–100% were revealed among the 31 ORF2 genes. In addition, the 31 isolates showed nucleotide identity of 48.0–99.9% and amino acid identity of 37.5–99.9% with 57 reference CAstV strains from GenBank (Supplementary Table S2).

**Figure 3:**
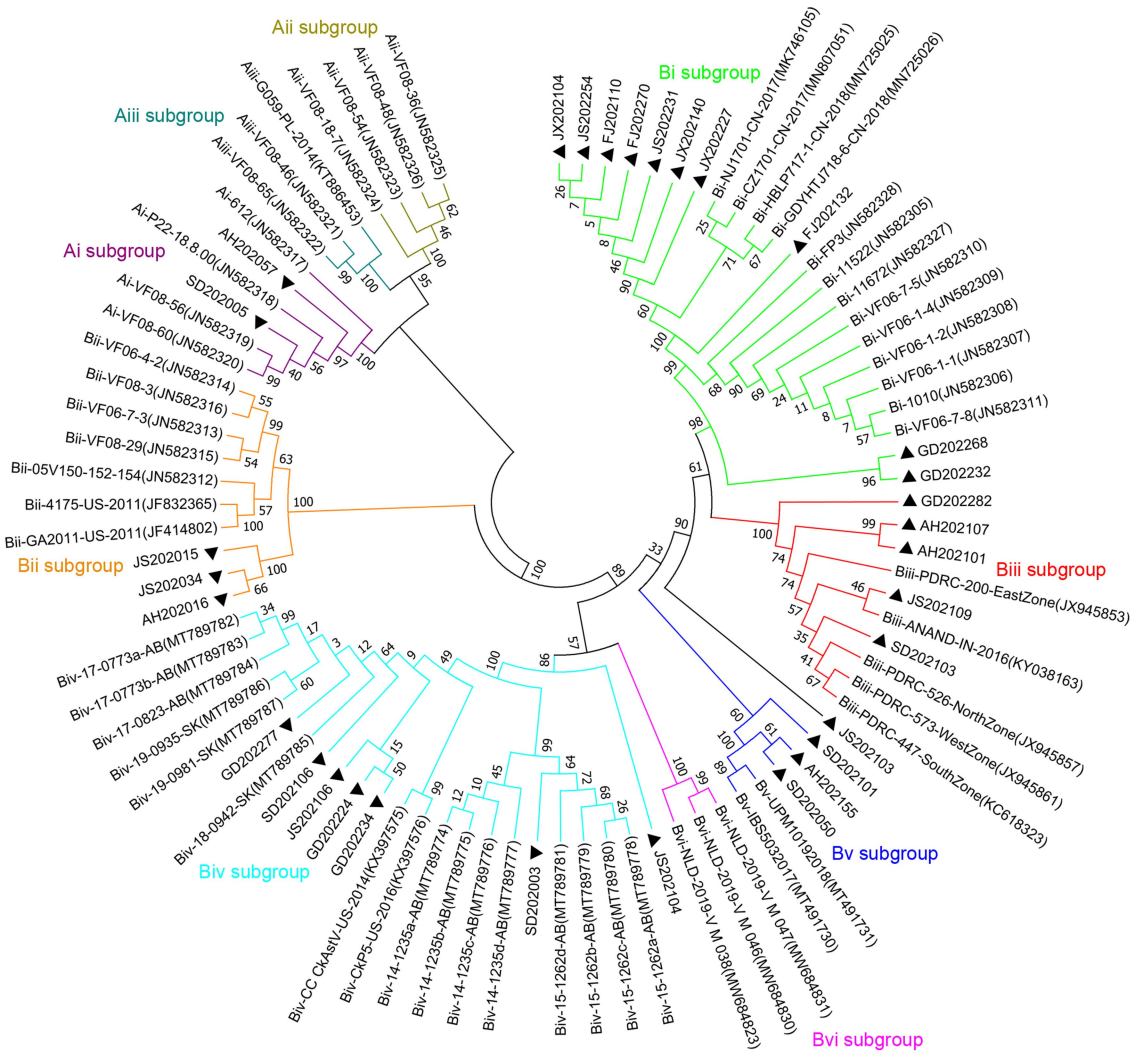
Phylogenetic tree of ORF2 amino acid sequences of 31 isolated strains (black solid triangles) and 57 reference strains using neighbor-joining method of the MEGA 7 0.0 software program with 1,000 bootstrap replicates. CAstV strains which belong to subgroup Ai are labelled with purple branches, CAstV strains which belong to subgroup Aii are labelled with dark-yellow branches, CAstV strains which belong to subgroup Aiii are labelled with dark-green branches, CAstV strains which belong to subgroup Bi are labelled with light-green branches, CAstV strains which belong to subgroup Bii are labelled with orange branches, CAstV strains which belong to subgroup Biii are labelled with red branches, CAstV strains which belong to subgroup Biv are labelled with light-blue branches, CAstV strains which belong to subgroup Bv are labelled with dark-blue branches, CAstV strains which belong to subgroup Bvi are labelled with pink branches.

For the phylogenetic analysis, the construction of a phylogenetic tree was performed according to ORF2 amino acid sequence of the 31 CAstV strains detected in the current work and the 57 reference strains. As displayed in Figure 3, among the 31 CAstV strains identified in our study, 2 isolates belong to subgroup Ai, 10 isolates belong to subgroup Bi, 3 isolates belong to subgroup Bii, 5 isolates belong to subgroup Biii, 7 isolates belong to subgroup Biv, and 3 isolates belong to subgroup Bv, with one isolate (JS202103) sharing a large cluster with other strains and belonging to a new B subgroup.

### Genome sequence and phylogenetic analysis of JS202103 strain

The whole genome sequence of the CAstV strain was derived by the combination of 7 overlapping cDNA fragments, and deposited at GenBank under accession number OR351273. The genome of the JS202103 strain was 7,507 nucleotide (**nt**) in length, including 5’-UTR (21 nt), ORF1a (3,423 nt), ORF1b (1,560 nt), ORF2 (2,217 nt), and 3’-UTR (281 nt) sequences. We compared JS202103 with 29 other CAstV strains available in GenBank (Supplementary Table S2), finding that the level of nucleotide identities ranged from 76.9 to 94.3% (Table 2). The highest genome sequence identity of 94.3% was observed between JS202103 isolate and four Canadian strains (14-1235a-AB, 14-1235b-AB, 14-1235c-AB, and 14-1235d-AB) belonging to subgroup Biv. The JS202103 strain had the least resemblance to one Poland strain G059/PL/2014 (subgroup Aiii) with 76.9% identity. The amino acid (**aa**) sequences of ORF1a and ORF1b of the JS202103 strain exhibited identities of 86.2–99.2% with another 29 CAstV strains (Table 2). However, the amino acid sequence of ORF2 of the JS202103 strain was highly similar (94.7%) to ANAND-IN-2016 strain (subgroup Biii) and lowly similar (41.1%) to G059/PL/2014 strain (Table 2).

**Table 2:** Comparisons of the genome, ORF-1a, ORF-1b, and ORF2 nucleotide and amino acid sequences of the JS202103 strain with 29 CAstV reference strains.

No.	Virus	GenBank number	Subgroup	Sequence similarities in percentages (%)							P-dist of ORF2 aa
				Genome	ORF1a nt	ORF1a aa	ORF1b nt	ORF1b aa	ORF2 nt	ORF2 aa	
1	G059/PL/2014	KT886453	Aiii	76.9	87.3	94.6	89.9	96	53.2	41.1	0.917
2	HBLP717/1/CN/2018	MN725025	Bi	80.7	77.1	87.4	78.5	86.3	86.2	92.7	0.078
3	GDYHTJ718/6/CN/2018	MN725026	Bi	81	77.6	87.2	78.2	86.2	86.1	92.8	0.076
4	CZ1801/CN/2018	MN807051	Bi	80.5	77.4	87.3	78.5	86.7	85.7	92.8	0.076
5	NJ1701/CN/2017	MK746105	Bi	80.8	77.3	87	78.8	86.7	86.4	92.8	0.076
6	4,175/US/2011	JF832365	Bii	88.2	92.9	96.8	93.6	88	76.9	83.2	0.162
7	GA2011/US/2011	JF414802	Bii	89.3	92.5	97.9	95.6	99.2	79.4	87.1	0.125
8	ANAND/IN/2016	KY038163	Biii	92.5	92.8	97.3	94.2	98.1	91.1	94.7	0.055
9	CC_CkAstV/US/2014	KX397575	Biv	92	93.5	97.6	93.3	98.5	87.9	93.5	0.062
10	CkP5/US/2016	KX397576	Biv	92.1	93.7	97.9	93.2	98.5	88	93.6	0.060
11	14/1235a/AB/2014	MT789774	Biv	94.3	97.6	98.5	96	98.5	87.6	93.2	0.060
12	14/1235b/AB/2014	MT789775	Biv	94.3	97.5	98.4	96	98.7	87.6	93.2	0.060
13	14/1235c/AB/2014	MT789776	Biv	94.3	97.6	98.5	96	98.5	87.6	93.2	0.060
14	14/1235d/AB/2014	MT789777	Biv	94.3	97.5	98.5	95.9	98.5	87.6	93.2	0.060
15	15/1262a/AB/2015	MT789778	Biv	94.2	97.4	98.2	96	98.5	87.4	93	0.063
16	15/1262b/AB/2015	MT789779	Biv	94.2	97.4	98.3	96	98.3	87.4	93	0.063
17	15/1262c/AB/2015	MT789780	Biv	94.2	97.4	98.3	96	98.5	87.3	93	0.063
18	15/1262d/AB/2015	MT789781	Biv	94.2	97.4	98.3	96	98.3	87.4	93.1	0.062
19	17/0773a/AB/2017	MT789782	Biv	91.1	91.5	96.2	94.2	98.5	87.4	92.7	0.070
20	17/0773b/AB/2017	MT789783	Biv	91.1	91.6	96.4	94	98.3	87.4	92.7	0.070
21	17/0823/AB/2017	MT789784	Biv	91.1	91.6	96.4	94.1	98.5	87.5	92.7	0.070
22	18/0942/SK/2018	MT789785	Biv	91.2	92.1	97.2	93.8	98.5	87.3	93.2	0.067
23	19/0935/SK/2019	MT789786	Biv	90.8	91.1	96.1	94	98.3	87.2	92.6	0.074
24	19/0981/SK/2019	MT789787	Biv	90.8	91.9	97.5	92.5	97.9	87.3	92.6	0.072
25	IBS5032017	MT491730	Bv	89.9	94.9	98.7	94.3	97.7	83	92.3	0.076
26	UPM10192018	MT491731	Bv	90.1	94.9	98.6	94.3	97.7	83	92.3	0.076
27	NLD-2019-V_M_038	MW684823	Bvi	91	94.8	98.4	94.5	97.5	81.9	90.1	0.101
28	NLD-2019-V_M_046	MW684830	Bvi	90.7	94.4	98.4	94	97.5	82.4	90.3	0.099
29	NLD-2019-V_M_047	MW684831	Bvi	90.8	94.5	98.4	94	97.5	82.4	90.3	0.099

**Figure 4:**
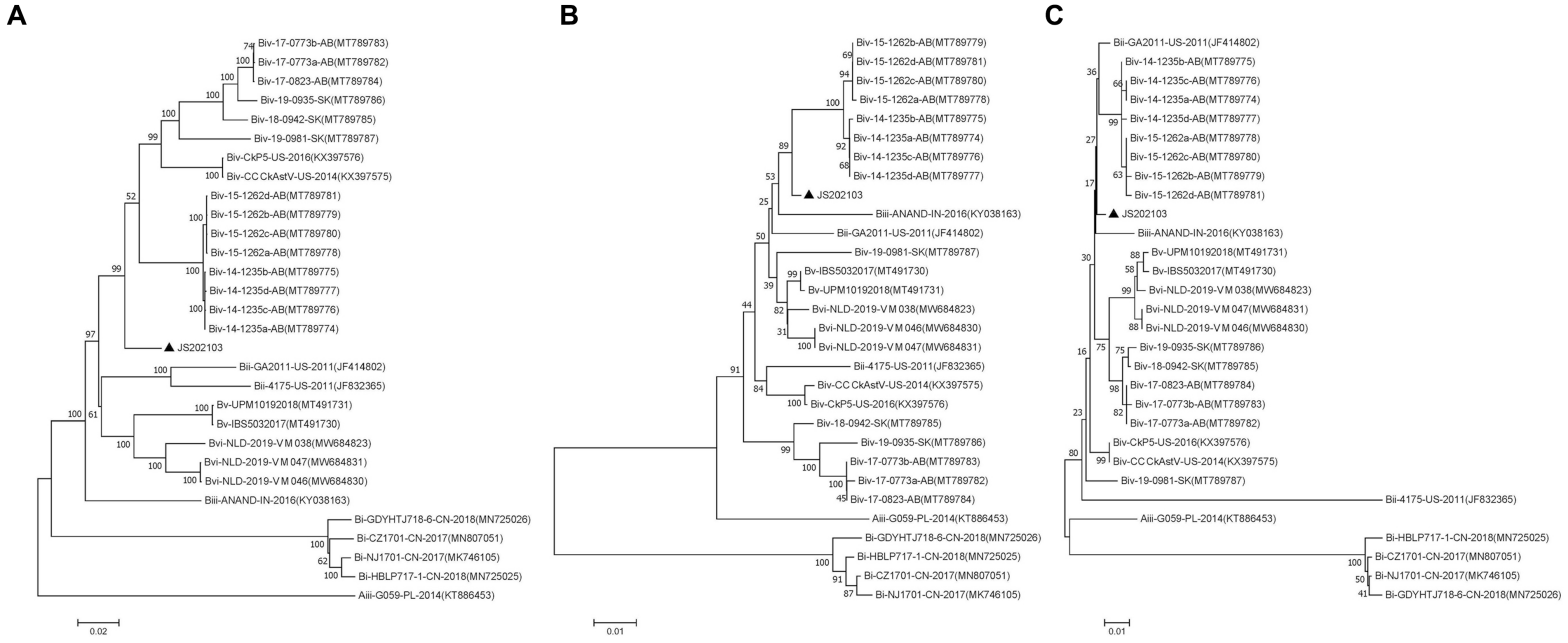
Phylogenetic analyses performed on the basis of the nucleotide sequences of full-length genomes **(A)**, amino acid sequences of ORF1a **(B)**, and amino acid sequences of ORF1b **(C)** of the CAstV strain JS202103 with the neighbor-joining method in MEGA 7 0.0 software program (1,000 bootstrap replicate). A black solid triangle was used to label new isolate.

Phylogenetic trees were constructed for the complete genome and specific proteins (ORF1a and ORF1b) of JS202103 and 29 reference strains. The phylogenetic tree was based on the whole genome, ORF1a and ORF1b protein indicated that the JS202103 was more tightly associated with the subgroup Biv strains (Figures 4A–C). However, the phylogenetic tree which was on the basis of ORF2 protein demonstrated that the JS202103 strain belonged to a new B subgroup (Figure 3). A whole genome recombination analysis between the JS202103 strain and 29 reference strains was performed using RDP4 and SimPlot software, and no recombination event was discovered in the examined samples.

### Pathogenicity of CAstV JS202103 isolate in chicken embryos

**Figure 5:**
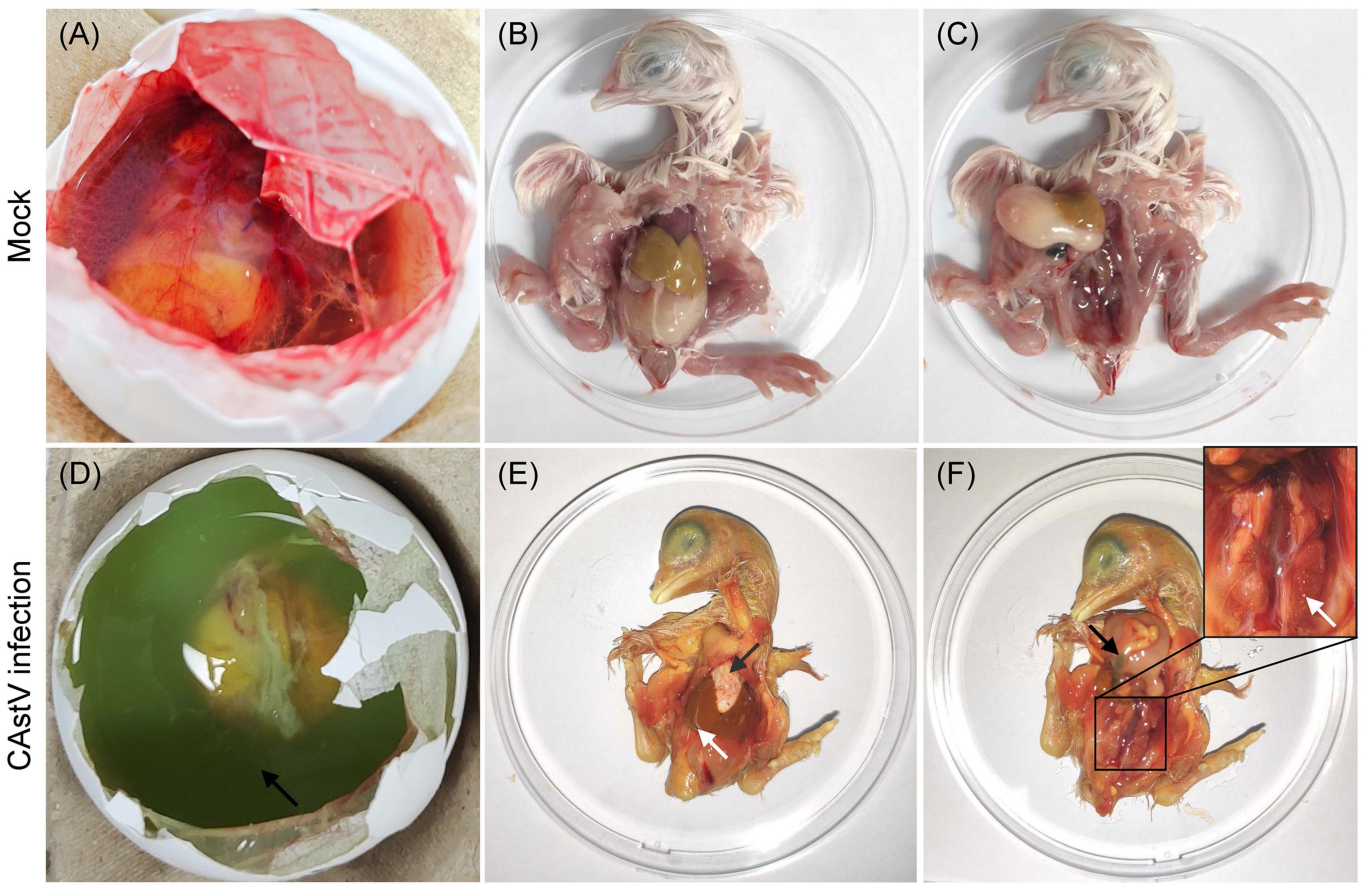
Macroscopic lesions from JS202103-inoculated and negative control 17 days-old chicken embryos. **(A–C)** No gross lesions were found in control chicken embryos. **(D)** The presence of green colour in the allantoic fluid. **(E)** The heart is covered with extensive white urate deposits (black arrow), and white thin membranous covering of the liver (white arrow). **(F)** Congested and enlarged kidneys with deposition of urate crystals (white arrow), and intestines filled with yellow and greenish content (black arrow).

For CAstV strain JS202103 from passage four, its pathogenicity was shown in SPF embryonated eggs. In the negative control group, the hatching rate was 100%, and all embryos exhibited no lesions (Figures 5A–C). Nevertheless, in the infected group, all embryos (100%) were found dead between 5 and 8 days post-infection. In addition, the main post-mortem results of dead-in-shell embryos were the existence of green colour in allantoic fluids (Figure 5D), hearts covered with extensive white urate deposits, white thin membranous covered with the liver (Figure 5E), congested and enlarged kidneys with deposition of urate crystals, and intestines filled with yellow and greenish content (Figure 5F).

**Figure 6:**
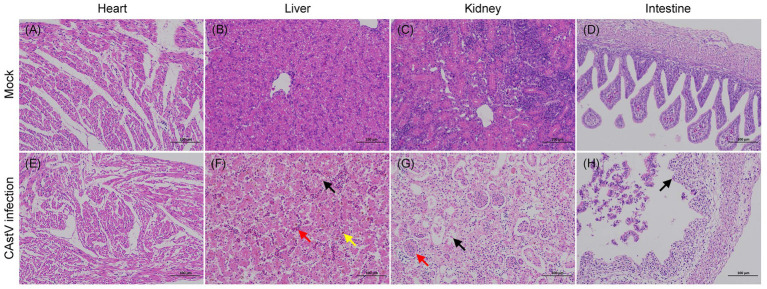
Haematoxylin and eosin-stained tissue sections from JS202103-inoculated and negative control 17 days-old chicken embryos. **(A–D)** Microscopic picture of a control chicken embryo. **(E–H)** Microscopic picture of a CAstV-infected chicken embryo. **(F)** Mild vacuolar degeneration of hepatocytes, small vacuoles in the cytoplasm (black arrow), mild congestion of hepatic sinuses (red arrow), and some brownish-yellow substance deposition (yellow arrow) were observed in the liver. **(G)** Extensive destruction of renal tubules, detachment of tubular epithelial cells, karyopyknosis and deep staining (black arrow), unclear glomerular capillary plexus as well as decreased cells (red arrow) in the kidney. **(H)** Extensive shedding of intestinal mucosa epithelium and exposed intestinal lamina propria were found in the intestine.

In addition, histopathological examination showed that microscopic lesions were found in the liver, kidney, and intestine of infected chicken embryos (Figure 6). In the liver, mild vacuolar degeneration of hepatocytes, small vacuoles in the cytoplasm (black arrow), mild congestion of hepatic sinuses (red arrow), and some brownish-yellow substance depositions (yellow arrow) were observed (Figure 6F). Extensive destruction of renal tubules, detachment of tubular epithelial cells, karyopyknosis and deep staining (black arrow), unclear glomerular capillary plexus and decreased cells (red arrow) were observed in the kidney (Figure 6G). Extensive shedding of intestinal mucosa epithelium and exposed intestinal lamina propria were found in the intestine (Figure 6H). The microscopic analysis of the heart did not show any alterations in the infected chicken embryos (Figure 6E), and the chicken embryos of the control group exhibited normal histopathology (Figures 6A–D).

**Figure 7:**
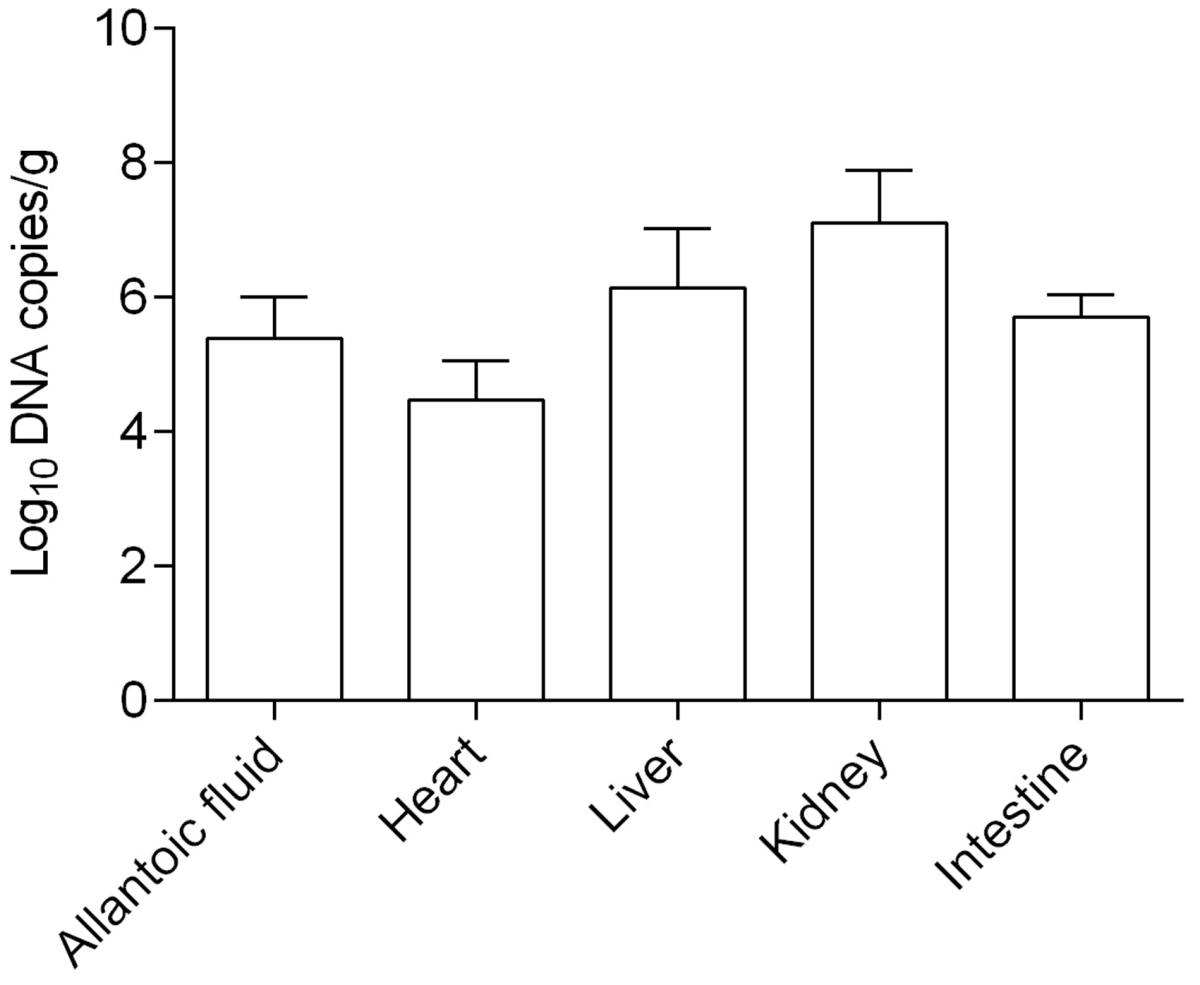
Viral load in allantoic fluids and various tissue samples of CAstV-infected SPF chicken embryos (*n* = five embryos).

In addition, the viral loads in different samples including allantoic fluid, heart, liver, kidney, and intestine were determined. As shown in Figure 7, the concentrations of the virus were in the range between 10^4.48^ and 10^6.15^ copies/g, except for the kidneys in which concentrations were higher (10^7.11^ copies/g). Obviously, the CAstV JS202103 strain showed extensive distribution within diverse tissues, while mostly concentrated in chicken kidneys. In addition, no CAstV RNA was found in tissue samples or allantoic fluid from control chicken embryos.

## Discussion

The extensively distributed astroviruses could be detected from various animal species, which included chickens, ducks, wild birds, and most mammals, as well as from humans, resulting in substantial financial losses in the poultry industry and bringing a serious threat to the health of human beings (Kurtz and Lee, 1987; Cortez et al., 2017). Since the initial identification of chicken astrovirus in broiler chicken flocks in 2004 in the Netherlands (Baxendale and Mebatsion, 2004), new strains of CAstV have been detected and featured in chicken flocks across the world (Sajewicz-Krukowska and Domanska-Blicharz, 2016; Palomino-Tapia et al., 2020; Yin et al., 2021; Raji et al., 2022). CAstV strains could be transmitted horizontally within chicken flocks via the faecal-oral route, and some strains may be vertically transmitted, causing hatchery diseases (McIlwaine et al., 2021; Raji et al., 2022). Recently, more and more clinical cases of CAstV infection are found in China (Xue et al., 2017; Zhao et al., 2020, 2021; Luan et al., 2022; Zhang et al., 2022). Nevertheless, there exists little information concerning the systematic epidemiology of CAstV infection. In this study, the epidemiology of 31 CAstV isolates between 2020 and 2022 in China was investigated. A new subgroup B CAstV strain JS202103 was successfully isolated from a 1 day-old chicken flock with poor viability of hatched chicks and growth problem in Jiangsu province and exhibited high pathogenicity to chicken embryos.

According to previous studies, multiple enteric viral infections are detected regularly, causing substantial financial losses in poultry industry worldwide (Kaithal et al., 2016; De la Torre et al., 2018; Chen et al., 2022). For example, the molecular examination of seven enteric virus of commercial chicken flocks in Brazil between 2010 and 2017 showed that 86.3% samples were positive for a single virus, and the remaining 13.7% were found in multiple infections (De la Torre et al., 2018). In addition, the detection and molecular characterization of seven enteric virus in poultry flocks in China indicated that 73.1% samples were single infection, multiple infections were identified in the remaining 26.9%, as well as 69% ChPV was single infection, whereas ANV (61.4%) and CAstV (56.1%) were multiple infections (Chen et al., 2022). According to the current epidemiological investigation, co-infections of CAstV with ChPV or ANV were also detected frequently, and 61.3% of CAstV was related to a single infection, whereas ChPV (22.6%) and ANV (16.1%) were co-infected with CAstV. Both ANV and ChPV infections were correlated with enteric disease, uneven growth, and runting-stunting syndrome (Kang et al., 2012; Zsak et al., 2013), which could probably worsen the clinical signs of CAstV in chicken flocks.

For the purpose of isolating CAstV, a stable leghorn male hepatoma cell line (LMH cells) was frequently adopted (Baxendale and Mebatsion, 2004; Kang et al., 2018; Zhao et al., 2020). In our study, we aimed to isolate virus from the 19 only CAstV-positive samples with LMH cells. The virus could only be isolated by inoculation with fresh homogenate in chickens. In addition, the difficulty in isolating CAstV from positive samples could probably be correlated with the fact that positive samples featured by real-time PCR might include noninfectious or low amount of virus. Even though there were many CAstV-positive samples using real-time PCR, only JS202103 was isolated, suggesting that there was extremely low success rate of isolation CAstV strains. As a result, the future studies are necessary in order to improve CAstV isolation.

As the most divergent region of the CAstV genome, ORF2 gene encodes capsid precursor proteins of the viral particle, which is the major determinant of virus antigenicity, immune response, cell tropism and viral pathogenicity (Smyth et al., 2012; Raji and Omar, 2022a,b). CAstV strains, based on phylogenetic analysis and the genetic distance of the ORF2 amino acid sequences, can be divided into two distinct genogroups (A and B): Group A with three subgroups (Ai, Aii, and Aiii), and Group B consists of six subgroups (Bi, Bii, Biii, Biv, Bv, and Bvi) (Smyth, 2017; Raji et al., 2022; Raji and Omar, 2022a,b). The previous studies have reported that diverse CAstV strains (subgroup Ai, subgroup Bi, subgroup Bii) are detected circulating in Chinese chicken flocks (Zhao et al., 2020; Luan et al., 2022). In this study, 31 CAstV isolates were characterized and analyzed, and sequence alignment based on the ORF2 amino acid showed that the 31 CAstV isolates shared 37.5–99.9% similarities with the 57 CAstV reference strains. The phylogenetic analysis which was based on the amino acid of ORF2 indicated that the 31 CAstV isolates fell into 7 subgroups, two isolates were subgroup Ai, ten isolates were subgroup Bi, three isolates were subgroup Bii, five isolates were subgroup Biii, seven isolates were subgroup Biv, three isolates were subgroup Bv, and one isolate (JS202103) belonged to a new B subgroup. This study is the first report that diverse CAstV strains in subgroups Biii, Biv, and Bv circulating in China.

As a ubiquitous enteric virus, CAstV is frequently isolated from the gut of clinically sick and healthy chickens (Shah et al., 2016; Kang et al., 2018; Kwoka et al., 2021; Raji and Omar, 2022a,b). Although some CAstV strains are probably apathogenic, specific CAstV strains can cause severe diseases, including uneven flock performance, runting-stunting syndrome, kidney disease, urate deposits and visceral gout, as well as white chick hatchery disease (Sajewicz-Krukowska and Domanska-Blicharz, 2016; Kang et al., 2018; Palomino-Tapia et al., 2020; Raji et al., 2022). For example, the FP3 strain of subgroup Bi, the Indian strains of subgroup Biii, and the Malaysian strains of subgroup Bv are reported to cause kidney disease and visceral gout in chickens (Smyth et al., 2007; Bulbule et al., 2013; Patel et al., 2017; Raji et al., 2022). The two US strains (CkP5/US/2016 and CC_CkAstV/US/2014) belonging to subgroup Biv are shown to act as the aetiologic agent of the runting-stunting syndrome in broiler chickens (Kang et al., 2018). The white chick hatchery disease, caused by CAstV subgroup Ai, Aiii, Bi, Bii, and Biv, has been found in Poland, China, Canada, and Brazil (Smyth et al., 2013; Sajewicz-Krukowska et al., 2016; Palomino-Tapia et al., 2020; Luan et al., 2022). In accordance with the amino acid sequence of ORF2, the sequence alignments showed that JS202103 isolate had a high similarity with the ANAND-IN-2016 strain (subgroup Biii), but a phylogenetic analysis indicated that JS202103 isolate belonged to a new B subgroup. According to the chicken embryo infection experiment, JS202103 caused hatchability reduction, dead-in-shell embryos, the existence of green colour in allantoic fluids, enlarged kidneys, urate deposits, and visceral gout, as well as intestine lesions, which are similar to the observations in other CAstV infections (Bulbule et al., 2013; Sajewicz-Krukowska et al., 2016; Raji et al., 2022).

In summary, CAstV infection was predominant in chicken flocks in six provinces in China during 2020–2022. In addition, a novel CAstV strain (called JS202103) belonging to a new B subgroup was successfully isolated *in vitro*, and demonstrated to be highly virulent to chicken embryos. Moreover, this is also the first study which provides systematic epidemiological features of CAstV, and reporting diverse CAstV strains in subgroups Biii, Biv, and Bv circulating in China. Moreover, these data could extend the molecular epidemiology and pathogenicity of the CAstV strains in China.

## Data availability statement

The datasets presented in this study can be found in online repositories. The names of the repository/repositories and accession number(s) can be found below: NCBI-OR351273.

## Ethics statement

All animal care and use protocols were performed strictly in accordance with the “Guidelines for Experimental Animals” of the Ministry of Science and Technology (Beijing, China). The study was conducted in accordance with the local legislation and institutional requirements.

## Author contributions

XH: Writing – original draft, Writing – review & editing. LY: Writing – original draft, Data curation, Formal analysis, Software. XL: Data curation, Formal analysis, Investigation. HL: Data curation, Formal analysis, Investigation.
