# Supplementary material for: Molecular characterization of chicken astrovirus and pathogenicity of a novel isolate in China
Source: Front Microbiol. 2023 Dec 11;14:1280313. doi: 10.3389/fmicb.2023.1280313 (PMC10751203; doi:10.3389/fmicb.2023.1280313)
Supplement: Supplementary file 1 [file Table_1.pdf]

Supplementary Table S1. Primers are used for the detection of potential viruses in broiler flocks with growth problems.

Virus	Primer	Nucleotide sequence (5'-3')	Reference
CAstV	Forward	GCYGCTGCTGAAGAWATACAG	(Smyth et al., 2010)
	Reverse	CATCCCTCTACCAGATTTTCTGAAA	
	Probe	FAM-CAGAAGTCGGGCCC-MGB	
ANV	Forward	GTAAACCACTGGYTGGCTGACT	
	Reverse	TACTCGCCGTGGCCTCG	
	Probe	FAM-CAGCAACTGACTTTC-MGB	
IBV	Forward	GCTTTTGAGCCTAGCGTT	(Scott et al., 2006)
	Reverse	GCCATGTTGTCACTGTCTATTG	
	Probe	FAM-CACCACCAGAACCTGTCACCTC-BHQ1	
NDV	Forward	AGTGATTGTCTCGGACCTTC	(Mark et al., 2004)
	Reverse	CCTGAGGAGAGGCATTTGCTA	
	Probe	FAM-TTCTCTAGCAGTGGGACAGCCTGC-TAMRA	
avian reovirus	Forward	GGCCTMTCTAGCCACACCT	(Li et al., 2020)
	Reverse	TGGAGRTCGATTCGAGGTT	
	Probe	ROX-TTCTCGYATTACCGCCTTAGATCGT-BHQ2	
FAdV	Forward	AAAACTGAGACTTTCCCACAA	
	Reverse	AGATACCCTCCGAAGAACTAC	
	Probe	HEX-TCTCCCATATCATTTCCATGCCTCC-BHQ1	
avian rotavirus	Forward	TTGGACCAGTATTTCCTGCTG	(David et al., 2018)
	Reverse	TGGTATGAGCTGTTACCCTCAA	
ChpV	Forward	GCAACTAACCTGACCGTGTG	(Luis et al., 2018)
	Reverse	CCCGGATTCAGAACCAGTAT	

Supplementary Table S2. The 57 CAstV reference strains retrieved from the GenBank database were used in the analysis.

No.	Virus	Subgroup	GenBank number	Phylogenetic tree				References
				Whole genome	ORF1a	ORF1b	ORF2	
1	G059/PL/2014	Aiii	KT886453	√	√	√	√	(Sajewicz-Krukowska and Domanska-Blicharz, 2016)
2	HBLP717/1/CN/2018	Bi	MN725025	√	√	√	√	(Xue et al., 2020)
3	GDYHTJ718/6/CN/2018	Bi	MN725026	√	√	√	√	
4	CZ1801/CN/2018	Bi	MN807051	√	√	√	√	(Zhao et al., 2021)
5	NJ1701/CN/2017	Bi	MK746105	√	√	√	√	
6	4175/US/2011	Bii	JF832365	√	√	√	√	N.A.
7	GA2011/US/2011	Bii	JF414802	√	√	√	√	(Kang et al., 2018)
8	ANAND/IN/2016	Biii	KY038163	√	√	√	√	(Patel et al., 2017)
9	CC_CkAstV/US/2014	Biv	KX397575	√	√	√	√	(Kang et al., 2018)
10	CkP5/US/2016	Biv	KX397576	√	√	√	√	
11	14/1235a/AB/2014	Biv	MT789774	√	√	√	√	(Palomino-Tapia et al., 2020)
12	14/1235b/AB/2014	Biv	MT789775	√	√	√	√	
13	14/1235c/AB/2014	Biv	MT789776	√	√	√	√	

14	14/1235d/AB/2014	Biv	MT789777	√	√	√	√	
15	15/1262a/AB/2015	Biv	MT789778	√	√	√	√	
16	15/1262b/AB/2015	Biv	MT789779	√	√	√	√	
17	15/1262c/AB/2015	Biv	MT789780	√	√	√	√	
18	15/1262d/AB/2015	Biv	MT789781	√	√	√	√	
19	17/0773a/AB/2017	Biv	MT789782	√	√	√	√	
20	17/0773b/AB/2017	Biv	MT789783	√	√	√	√	
21	17/0823/AB/2017	Biv	MT789784	√	√	√	√	
22	18/0942/SK/2018	Biv	MT789785	√	√	√	√	
23	19/0935/SK/2019	Biv	MT789786	√	√	√	√	
24	19/0981/SK/2019	Biv	MT789787	√	√	√	√	
25	IBS5032017	Bv	MT491730	√	√	√	√	(Raji et al., 2022)
26	UPM10192018	Bv	MT491731	√	√	√	√	
27	NLD-2019-V_M_038	Bvi	MW684823	√	√	√	√	
28	NLD-2019-V_M_046	Bvi	MW684830	√	√	√	√	(Kwoka et al., 2021)
29	NLD-2019-V_M_047	Bvi	MW684831	√	√	√	√	
30	612	Ai	JN582317				√	(Smyth, V. J. 2017)

31	P22-18.8.00	Ai	JN582318	√
32	VF08-56	Ai	JN582319	√
33	VF08-60	Ai	JN582320	√
34	VF08-54	Aii	JN582323	√
35	VF08-18-7	Aii	JN582324	√
36	VF08-36	Aii	JN582325	√
37	VF08-48	Aii	JN582326	√
38	VF08-46	Aiii	JN582321	√
39	VF08-65	Aiii	JN582322	√
40	11522	Bi	JN582305	√
41	1010	Bi	JN582306	√
42	11672	Bi	JN582327	√
43	FP3	Bi	JN582328	√
44	VF06-1-1	Bi	JN582307	√
45	VF06-1-2	Bi	JN582308	√
46	VF06-1-4	Bi	JN582309	√
47	VF06-7-5	Bi	JN582310	√

48	VF06-7-8	Bi	JN582311	√	
49	05V150-152-154	Bii	JN582312	√	
50	VF06-7-3	Bii	JN582313	√	
51	VF06-4-2	Bii	JN582314	√	
52	VF08-29	Bii	JN582315	√	
53	VF08-3	Bii	JN582316	√	
54	PDRC-447-SouthZone	Biii	KC618323	√	N.A.
55	PDRC-200-EastZone	Biii	JX945853	√	
56	PDRC-526-NorthZone	Biii	JX945857	√	
57	PDRC-573-WestZone	Biii	JX945861	√	
